# Supplementary material for: Splice-Junction-Based Mapping of Alternative Isoforms in the Human Proteome
Source: Cell Rep. Author manuscript; Available in PMC 2020 Jan 15. (PMC6961840; doi:10.1016/j.celrep.2019.11.026)
Supplement: 3 [file NIHMS1546469-supplement-3.zip › DF2/PXD0010154/Esophagus-221-P11532-QTVLEGDNMETPASSPQLSHDDTHSR.pdf]

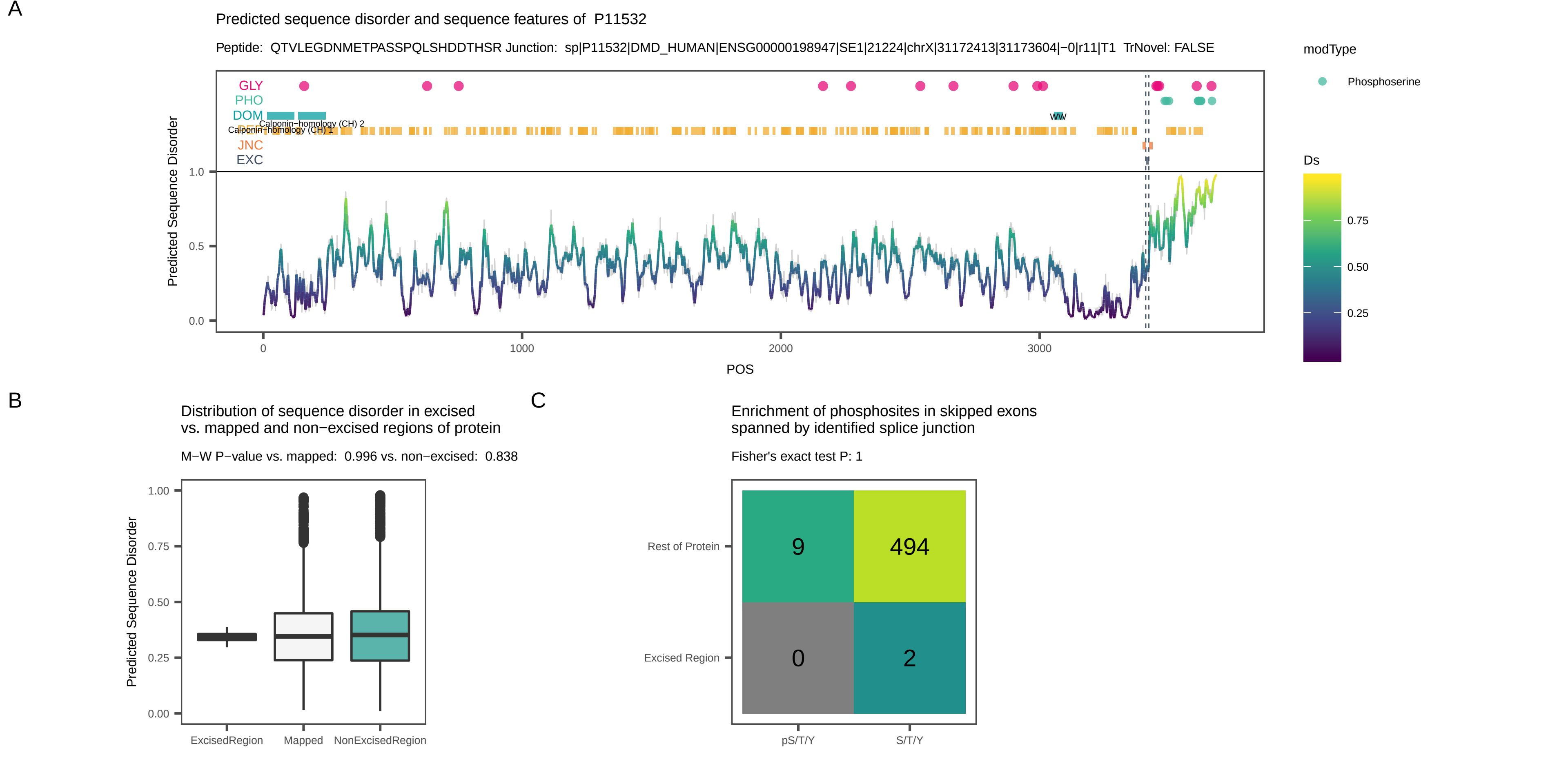
A
Predicted sequence disorder and sequence features of P11532
Peptide: QTVLEGDNMETPASSPQLSHDDTHSR Junction: sp|P11532|DMD_HUMAN|ENSG00000198947|SE1|21224|chrX|31172413|31173604|−0|r11|T1 TrNovel: FALSE
GLY
PHO
DOM
JNC
EXC
Calponin−homology (CH) 2
Calponin−homology (CH) 1
WW
Predicted Sequence Disorder
1.0
0.5
0.0
0
1000
2000
3000
POS
modType
Phosphoserine
Ds
0.75
0.50
0.25
B
Distribution of sequence disorder in excised
vs. mapped and non−excised regions of protein
M−W P−value vs. mapped: 0.996 vs. non−excised: 0.838
Predicted Sequence Disorder
1.00
0.75
0.50
0.25
0.00
ExcisedRegion
Mapped
NonExcisedRegion
C
Enrichment of phosphosites in skipped exons
spanned by identified splice junction
Fisher's exact test P: 1
Rest of Protein
Excised Region
9
494
0
2
pS/T/Y
S/T/Y
